# Construct validity and factor structure of sense of coherence (SoC-13) scale as a measure of resilience in Eritrean refugees living in Ethiopia

**DOI:** 10.1186/s13031-019-0185-1

**Published:** 2019-02-06

**Authors:** Berhanie Getnet, Atalay Alem

**Affiliations:** 1 Department of Psychiatry, College of Health Sciences, Addis Ababa University, Addis Ababa, Ethiopia; 2 Department of Psychology, University of Gondar, P.O. Box 196, Gondar, Ethiopia; 3 Amanuel Hospital, P.O.Box 9086, Addis Ababa, Ethiopia

**Keywords:** Sense of coherence, Resilience, Constructs validity, Factor structure, Eritrean refugees, Ethiopia

## Abstract

**Background:**

There is a scarcity of adapted measures to study resilience and mental health of people in humanitarian settings in Africa. The aim of this study was to identify the factor structure and other psychometric properties of the Sense of Coherence (SoC-13) scale in Eritrean refugees living in Ethiopia.

**Methods:**

In a cross-sectional survey, 562 adults were selected randomly from Eritrean refugees living in Mai Aini camp, Ethiopia. The SoC-13, the Center for Epidemiologic Studies Depression scale (CES-D), the Pre and Post-Migration Living Difficulties checklist, the Primary Care Post-Traumatic Stress Disorder screener (PC-PTSD), the Oslo Social Support Scale (OSS-3), the Coping Style scale and the Fast Alcohol Screening Test (FAST) were administered concurrently. Confirmatory Factor Analysis (CFA) was used to investigate the factor structure of the SoC-13 using IBM SPSS Amos, version 21.

**Result:**

A one factor model of the SoC with twelve items had the best fit to the current data (CFA = 0.982, RMSEA = 0.035 [90%CI = 0.018, 0.050]), with good internal consistency (Cronbach’s Alpha = 0.75). When all 13 items of the Tigrigna version were considered, there was an inverse association of SoC-13 with PC-PTSD(r = − 0.433, *p* < 0.001), CES-D (r = − 0.597, *p* < 0.001), Pre and post-migration living difficulties checklist (r = − 0.265, *p* < .001and r = − 0.249, p < 0.001 respectively), and FAST (r = − 0.105, *p* < 0.001), providing support for the divergent validity of the scale. The SoC-13 was associated positively with the Oslo Social Support scale (OSS-3)(r = 0.363 *p* < 0.001) and task-oriented coping (r = 0.089, p < 0.001), demonstrating convergent validity. The four items, specifically item-1, item-2, item-3 and item-12 have shown relatively weaker item loadings (β<0.40); but item-2 demonstrated non-significant loading (β = 0.06, p>0.05) in a one factor model of SoC-13.

**Conclusions:**

Although the 13-items of the Tigrigna version of the SoC scale loaded significantly onto their respective factors in the three factor model, only 12 items loaded significantly onto the one factor model, which demonstrated superior fit to the current data. Keeping in mind that future research should examine the conceptualizations of the four items demonstrating poor convergent validity in this Eritrean sample, the reduced Tigrigna version of SoC-12 is a reasonable measure of sense of coherence in this community.

**Electronic supplementary material:**

The online version of this article (10.1186/s13031-019-0185-1) contains supplementary material, which is available to authorized users.

## Study background

Mental health research is increasingly focusing on the importance of investigating protective factors such as coping style and resilience in people who are vulnerable to developing mental health problems in the context of a humanitarian crisis [1–3], as well as those with chronic physical health conditions [4–6]. Knowledge about such personal resources is vital for the purpose of making informed decisions while planning an intervention. Following the *salutogenic model* of human resilience, which is considered to be a paradigm shift from the adversities (pathogenic) model to the strengths model, understanding resilience is becoming the focus of research in humanitarian settings [7]. There is also a shift in attention to positive human functioning and the means of achieving an optimal level of wellbeing, although a number of research studies deal with the negative effect of trauma [8].

Resilience refers to the process of negotiating, managing and adapting to significant sources of stress or trauma [9]. Resilience is considered to be common and the normal response of people to conditions of adversity [7]. Unlike the pathogenic paradigm, which focuses on the aetiology of disease, the focus of the *salutogenic paradigm* is on sources of health, and hence it deals with the mechanisms which underlie management of stress to achieve health [6]. Among others, sense of coherence is a personality-focused collective attribute employed for predicting health, which is protective against the negative consequences of adverse events [10].The definition of sense of coherence provided by Antonvsky (1987, p.19) is: “a global orientation that expresses the extent to which one has pervasive, enduring though dynamic feelings of confidence that (1) the stimuli deriving from one’s internal and external environments in the course of living are structured and predictable and explicable; (2) the resources are available to meet the demands posed by the stimuli; and (3) these demands are challenges worthy of investment and engagement” [11]. There are three underlying constructs within sense of coherence. These include: *comprehensibility,* which refers to an enduring way of conceptualizing circumstances in an orderly, consistent, structured and clear way; *manageability* which refers to understanding the availability of adequate resources to cope with demands, whereas *meaningfulness* refers to the values that individuals place one vents irrespective of their effect, and which, therefore, deserve effort and commitment [12]. Although a number of studies employ the long version of Antonovsky’s SoC scale having twenty nine items (SoC-29), some studies have used the adapted version of the short form of the Sense of Coherence Scale with 13 items (SoC-13) to study resilience among adult forced migrants in humanitarian settings [1, 2, 13]. The validity and robustness of the SoC-13 scale is confirmed by its increased application in different places of the world, including: North America, Europe, Australia, South Africa, and the Middle East [2]. Although there is a debate in the literature regarding the extent to which sense of coherence and resilience are distinct concepts [14], the consensus is that sense of coherence is a comprehensive and over-arching concept, which includes resilience and hardiness [9].

Sense of coherence has been found to have a significant inverse relationship with mental health problems such as Post Traumatic Stress Disorder (PTSD) and depression [15], as well as adverse health conditions such as: rheumatoid arthritis [4], coronary heart disease [5] and congenital heart disease [6].

In a previous study amongst Eritrean internally displaced persons (IDPs), the sense of coherence scale (SoC-13) was found to have adequate psychometric properties to measure resilience [2].There are no consistent findings regarding the factor structure of SoC-13 when tested using Confirmatory Factor Analysis (CFA) across different cultures [16–18]. In this regard, a previously validated instrument is not guaranteed to remain valid in another time, culture or context [19]. For example, in a study examining the dimensionality of SoC-13 using CFA in an Italian sample, a one-factor model best fit their data [16]. In contrast, a three factor model of SoC-13 had a better fit than a single factor model in Peruvian college students [20]. In a systematic review of 458 scientific publications and 13 doctoral theses, it was reported that the factorial structure of the scale remain unclear, with some evidence supporting Antonovsky’s single factor solution while other evidence supported two or three factor solutions [17].

Although extensive studies on the topics of resilience and sense of coherence have been carried out among Eritreans in humanitarian settings [2, 7, 10, 21–23], empirical studies on the adaptation of resilience measures and their validity have not been given due attention. The SoC-13 has been employed to study resilience among displaced Eritreans in all nine languages of Eritrea [2]. Although the SoC-13 has been reported to be contextually appropriate for Eritrean culture and adequate to measure resilience in this population, the basis for this conclusion is qualitative evidence [2, 22]. There has been no quantitative investigation of the psychometric properties of the instrument in this community. Hence, the aim of the present study was to identify the factor structure and examine other psychometric properties of the SoC-13, including internal consistency, construct validity (divergent convergent and discriminant validity) and to address this evidence gap.

## Methods

### Materials and methods

#### Study settings and context

This study was conducted at Mai Aini refugee camp, one of the four camps in Northern Ethiopia for Eritrean refugees. The camp is situated at a distance of 1116 km to the north of Addis Ababa, the capital of Ethiopia. Mai Ani camp was established in 2008 by the United Nations High Commissioner for Refugees (UNHCR) [24]. As of 2013, this camp alone hosted about 17,825 Eritrean refugees [25]. Within the camp, there are three churches for Orthodox, Protestant and Catholic religious followers and one Mosque. Different humanitarian institutions provide health services in the camp. Among others, the two institutions that offer healthcare services are: the Administration of Refugees and Returnees Affairs (ARRA) health center and the Center for Victims of Trauma (CVT), the latter offering counseling and other forms of mental health care.. In addition, the Norwegian Refugee Council (NRC), International Rescue Committee (IRC) and Jesuit Refugee Service (JRC) provide education; psychosocial care and logistical support to Eritrean refugees [26]. Activities of these organizations are jointly run by a coordinated task of ARRA of Ethiopian government and UNHCR [27].

### Study design

The study was nested in a cross-sectional survey investigating mental health and sources of resilience in Eritrean refugees living in Ethiopia. In this paper, we examined the validity and psychometric properties of the adapted Tigrigna version of the Sense of Coherence (SoC-13) scale.

### Sample size and sampling procedures

In order to estimate the sample size, an average PTSD prevalence of 30.73% among refugees and forced migrants in East African camps [28–30] with 4% precision and 95% confidence was assumed. A further 10% was added to account for non-response, which resulted in a final sample size of 562.

In order to determine a sampling frame, first we obtained a document about registered refugees from the camp administration. According to findings from a census conducted by UNHCR, there were a total of 10,006 Eritrean refugees registered in Mai Aini camp in January 2016. However, this was incomplete and so we chose to undertake a rapid census of houses in Mai Aini camp. The census took two weeks to carry out. A total of 2055 houses were registered together with their house code numbers, of which 100 houses were excluded because they were occupied by minors (unaccompanied children living without their parents or guardians). The remaining 1955 houses for adults became a sampling frame, and from these, 562 houses were selected using simple random sampling using IBM SPSS, version 20. Finally, from each selected household, a single participant was selected using a lottery method from among eligible members of the household. Inclusion criteria for eligibility included the following: minimum of 18 years of age, Eritrean nationality before migrating to Ethiopia, having refugee status at the time their participation and being well enough to give consent and answer to the survey questions.

**Fig. 1:**
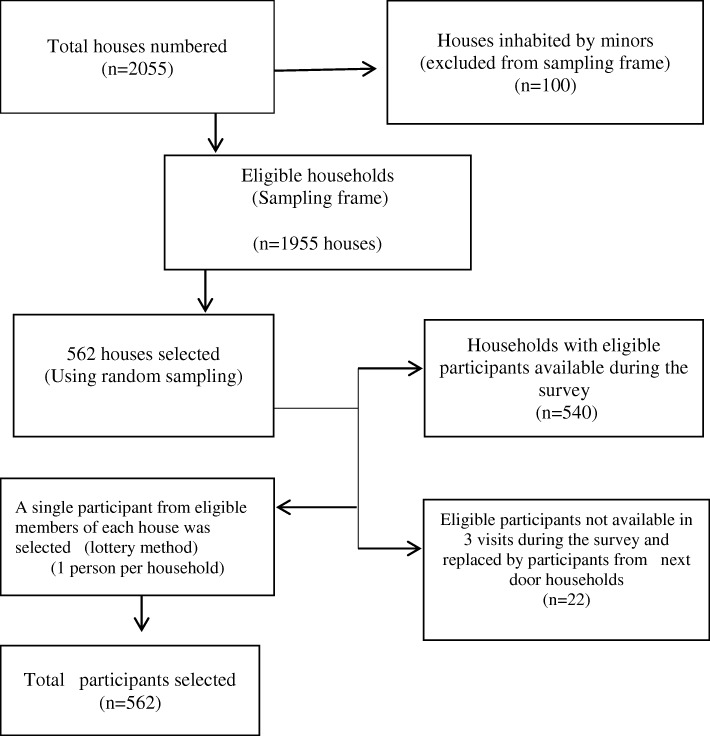
Flow chart depicting procedures of sampling

The dynamic settlement of refugees, even after conducting the census made it difficult to access all members in the randomly selected houses. Hence, twenty two houses (3.9% of the sample) were replaced by neighboring houses (i.e. from among those that preceded or followed the selected houses), because household members in the selected houses were not available around their home after three visits (see flow chart on procedures for sample selection in Fig. 1). Furthermore, in order to minimize missing data, the Principal Investigator (PI) provided on site supervision during data collection, and data collectors revisited households where items were missing.

### Adaptation procedures

All instruments were adapted following recommended procedures for trans-cultural study [31]. First, instruments were translated from the source language (English) into the target language (Tigrigna) by two bilingual experts, and then back translated into English by two independent bilingual translators who did not have the original version. Four experts in the field reviewed the translations and back translations, and then two consensus meetings were held in Addis Ababa and Mekele Universities. Items in the final version of the translations were then rated using a 4-point rating scale, with score values for ‘not relevant’ = 1, ‘somewhat relevant’ = 2, ‘quite relevant’ = 3, ‘highly relevant’ = 4 [32] for their content relevance by seven experts (i.e. one psychiatrist, five practitioners working in psychiatric wards and one clinical psychologist). The purpose of these ratings was to obtain a content validity index [32, 33].

Following this, cognitive interviews were then carried out to check the feasibility and understandability of each item in the instrument, and hence minor revisions were made based on respondents’ feedback. All the instruments were pilot tested before they were employed to collect data for the main study. Data collection took place from January to March, 2016 after providing sufficient training to data collectors.

### Measures

The following instruments were used:

*Sense of coherence Scale (SOC-13, Antonovsky, 1994)*

Resilience was measured using the *Sense of Coherence (SoC-13*) *scale* [34]. This is a brief 13-itemscale adapted to the Eritrean culture in the form of a 5-point Likert scale from the original 7-point scale to improve comprehensibility [35] (see Additional file 1: Table S3). The instrument was reported to have been adapted to the Eritrean culture, and has proved to be an adequate measure of resilience [2].

*Pre and Post Migration Living Difficulties Checklist (Idemudia, et al., 2013)*

This is a five point response format (i.e. strongly disagree scored 1; disagree = 2; neutral =3; agree = 4, and strongly agree =5) [36]. It was employed to measure pre and post migration difficulties of homeless Zimbabwean refugees in South Africa, and the instrument had shown good internal consistency in a pilot study up on homeless Zimbabweans [36].

*Center for Epidemiologic Studies Depression Scale (CES-D, Radloff, 1977)*

This is a 20 item brief scale with four alternative response options, with ranges from “None of the time” scored as 0 to “Most of the time” scored as 3 [37]. CES-D was translated and validated into Tigrigna language for Tigrigna speaking Eritrean refugees in the United States by Moges (2011), and found the internal consistency alpha value of 0.86 and test re-test reliability r = 0.91(*n* = 253) [38].

*Primary Care PTSD Screener (PC-PTSD, Prins et al., 2003)*

This is a four item brief PTSD screening instrument, having two option response levels to be responded as ‘Yes’ and ‘No’ [39]. Test re-test reliability was found to be 0.83 and it is reported to be good [39]. Furthermore, the sensitivity and specificity of PC-PTSD was found to be 0.78 and 0.87 respectively [39]. Besides the scale’s extensive use to study PTSD among veterans of United States [39, 40], it was also employed to study mental health of veterans in samples drawn from Iraq and Afghanistan [41]. Its use to study PTSD in refugees is also well documented [42, 43].

*Coping Style Scale, (Transcultural Psychosocial Organization, TPO)*

In order to measure the coping strategies, a list of 10 items was culturally validated and translated into Amharic in a 7 step procedure by Trans-cultural Psychosocial Organization (TPO), and later used to study displaced Ethiopians from Eritrea [44]. The items require participants to respond in terms of “this is not like me” or “this is like me” [44]. This scale roughly captured three coping strategies, including: task-oriented, avoidance-oriented and emotion-oriented coping strategies [44]. Since the instrument was employed to measure the coping styles in participants of displaced Ethiopians from Eritrea who share similar socio-cultural conditions with the target population of the present study, it was employed to measure coping strategies after making proper adaptation.

*Oslo Social support Scale (OSS-3, Dalgard et al., 2006))*

Refugees’ social support was measured using *Oslo Social support Scale (OSS-3)* [45]. This is a brief scale consisting of three items in which the sum score scale ranges from 3 to 14 [45]. This tool was adapted in an African context. For example, in a validation study of OSS-3 in Nigeria, the internal consistency Cronbach’s alpha value was found to be 0.5 [46]. There is an increased use of OSS-3 in an Ethiopian context. Most notably it was employed in a study which involved population levels in Rural Ethiopia [47].

*Fast Alcohol Screening Test (FAST, Hodgson, et al., 2002)*

Alcohol use was measured using *Fast Alcohol Screening Test (FAST*) [48]. FAST is a brief four items tool meant to measure alcohol use, which was derived from taking few items from Alcohol Use Disorder Identification Test (AUDIT) [48, 49]. Each item is scored from 0 to 4, whose total score was considered FAST positive for total scores > 3 [48]. Test-retest reliability of the total score for inter-rater agreement was 0.83, demonstrating excellent agreement [49]. FAST has demonstrated overall sensitivity (91%) and specificity (93%) [48]. The FAST was recommended to be in use in busy medical centers [49]. Population based studies used this tool in the settings of East Africa, including Ethiopia [47].

### Statistical analysis

Confirmatory Factor Analysis (CFA) was employed to determine the best fitting model to the present data from alternative models of SoC-13 underlying the construct of sense of coherence in literature. Before running CFA analysis, basic assumptions with respect to sampling adequacy, possibilities for violations of multi-co linearity and normality of data was evaluated using the Kaiser-Meyer-Olkein measure of sampling adequacy, Durbin Watson test and box plot, respectively. CFA is a measure to compare data with theoretical model [50]. In evaluating a model, we used indices of acceptable fit, specifically values for the ratio of chi-square over degree of freedom (χ^2^/df) to be 3:1or less indicates good fit; Comparative Fit Index(CFI) close to 0.95, Root Mean Square Error of Approximation(RMSEA) close to 0.06 and Standardized Root Mean Residual (SRMR) close to 0.06 [50]. Furthermore, Content validity was analyzed by Content Validity Index(CVI), with estimates for item level content validity index (I-CVI) as well as scale level content validity index (S-CVI) for content relevance [33].I-CVI refers to the proportion in the number of experts who give 3 and 4 for a given item for its content relevance in relation to the total number of experts who rated the scale, whereas S-CVI indicates the proportion in the number of items which were given 3 and 4 in relation to the total number of items in the scale [32, 51].

Among the two methods of scale level content validity index, the average calculation method (S-CVI/Ave) is expected to be greater than or equal to 0.90 [52]. The proportion of agreement on the relevance of each item (I-CVI) should be at least 0.78 [32, 33]. Convergent validity was assessed by examining the extent to which the indicators loaded onto the expected factors; divergent or discriminate validity was judged using the correlation between the latent factors [53]. Discriminant validity is considered adequate when this correlation is less than or equal to 0.80 or 0.85 [53].

## Result

### Demographic characteristics of participants

Of the 562 participants, 304 (54.1%) were females. Ages ranged from 18 to 74 (mean = 29.63, SD = 10.18); the vast majority were literate; the average years of stay in the refugee camp was 3.71 years, and the great majority of the participants belonged to the Tigriya ethnic group (92%). Very few participants came from Saho, Bilen, Tigre and Jabelty ethnic groups of Eritrea constituting 8% altogether. When it comes to religion, 84% were followers of Orthodox Christianity. The study participants had a diverse profile of occupations before coming to Ethiopia; 71% constituted students, military and farmers (see Table 1).

**Table 1:** The demographic characteristics of participants

Characteristics		Number (%)
Sex	Male	258 (45.9)
	Female	304 (54.1)
Age	Mean(SD)	29.6 (10.2)
	18–24	205 (36.5)
	25–34	219 (39.0)
	35–44	89 (15.8)
	45–54	29 (5.2)
	55–64	15 (2.5)
	65–74	5 (0.9)
Educational Background	Non-literate	67 (11.9)
	Elementary school	232 (41.3)
	Secondary school	238 (42.3)
	College graduate or above	25 (4.5)
Marital status	Single	189 (33.6)
	Married	327 (58.2)
	Divorced	29 (5.2)
	Widowed	17 (3.0)
Religion	Orthodox	477 (84.9)
	Protestant	17 (3.0)
	Catholic	23 (4.1)
	Muslim	44 (7.8)
	Jehovah witness	1 (0.2)
Past occupation in Eritrea	Student	201 (35.7)
	Military	111 (19.8)
	Farmer	89 (15.8)
	Home maid	66 (11.75)
	Educator	23 (4.1)
	Daily laborers	15 (2.7)
	Others	57 (13.1)

### Internal consistency of sense of coherence (SoC-13 items)

The internal consistency Cronbach’s alpha values of SoC-13 for the pilot study (*n* = 52) and main study (*n* = 562) were found to be 0.67 and 0.74, respectively. When the three theoretical sub-scales were tested for their internal consistency, the corresponding Cronbach’s alpha values for main study in each sub-scale diminished to 0.56 or less compared to the total 13-items, which resulted in 0.74 (see Additional file 2: Table S1).The internal consistency improved a bit higher (Cronbach’s Alpha value > 0.74) on a condition that item1, item-2 and item-12 were discarded (see Additional file 3: Table S2).The internal consistency for the twelve items (except for item-2) resulted in Cronbach’s alpha value of 0.75, and hence internal consistency remained stable with the omission of a single item.

### Content validity

The Item Level Content Validity Index (I-CVI) in the present study ranged from 0 .86 (item-5) to 1 for the rest of the12 items (see Additional file 3: Table S2). The Average of Scale Level Content Validity Index (S-CVI/ Ave) for the total scale resulted in 0.989. Both the S-CVI and S-CVI/Ave are above the lowest threshold value of scale level content validity index for SoC-13 in the present study.

### Confirmatory factor analysis (CFA)

In order to test the factor structures of SoC-13 using CFA, first, the assumptions needed to run factor analysis were tested. Thus, Kaiser-Meyer-Olkin (KMO) test for measure of sampling adequacy showed 0.820, and Chi square for Bartlett’s test of sphericity was significant (χ^2^ = 1467.7,df = 78, *p* < 0.001). In addition, the minimum sample size for factor analysis, which requires > 200 was met (*n* = 562).

Examination of the present data against plausible computing measurement models of SoC-13 indicated that the three correlated factors model of SoC-13, with correlated error terms, demonstrated poor fit (CFI = 0.786; RMSEA = 0.098).

**Fig. 2:**
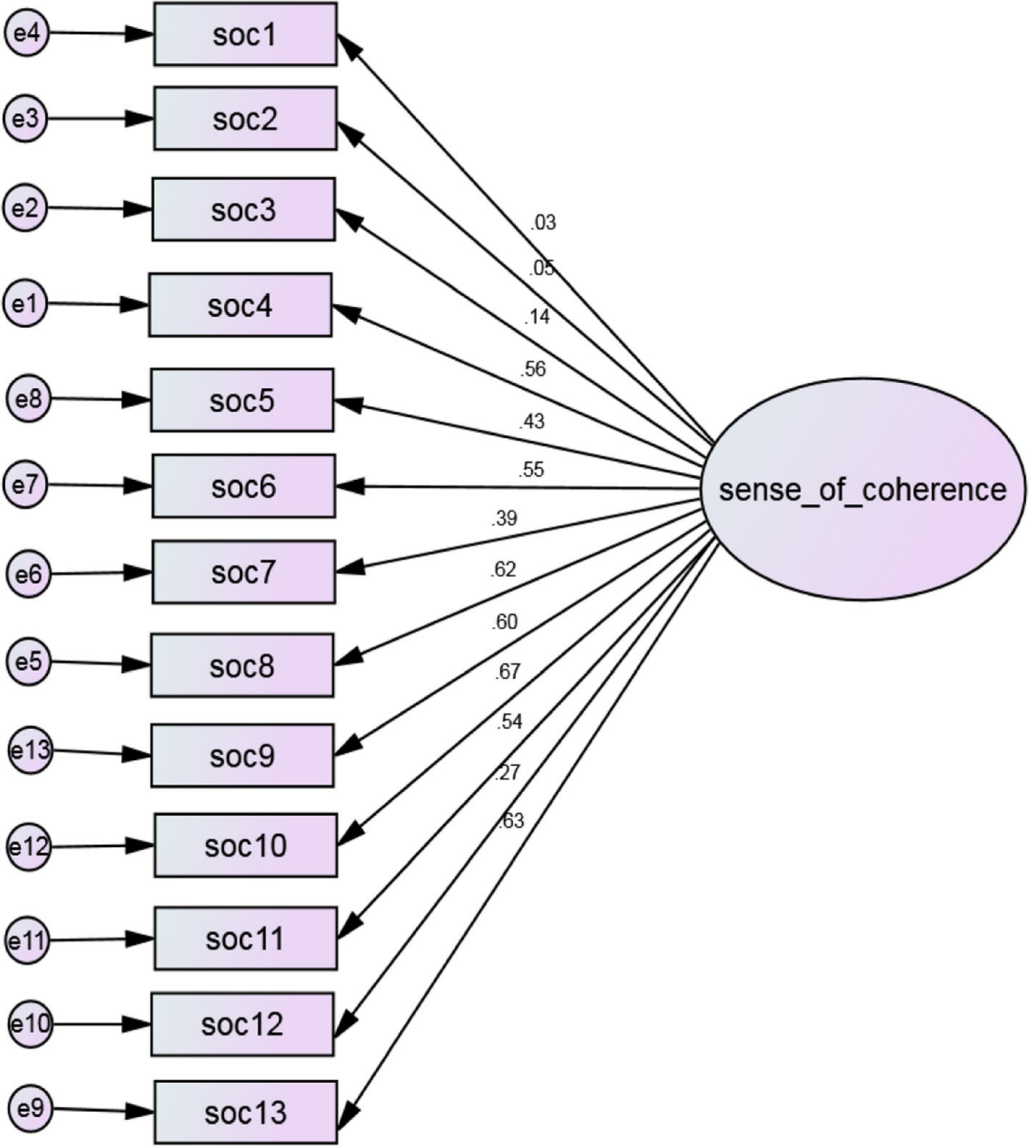
One factor model of SoC-13, with uncorrelated error terms.Rectangles represent indicator items; ovals represent latent factors; single headed arrows along with standardized weights represent factor loadings;circles represent error terms (e) for each item. Model fit: x2=412.363; df=65; x2/df=6.34; CFI= 0.752; TLI= 0.703; GFI= 0.889; RMSEA= 0.098 (90%CI: 0.089, 0.107); SRMR= 0 .0791

**Fig. 3:**
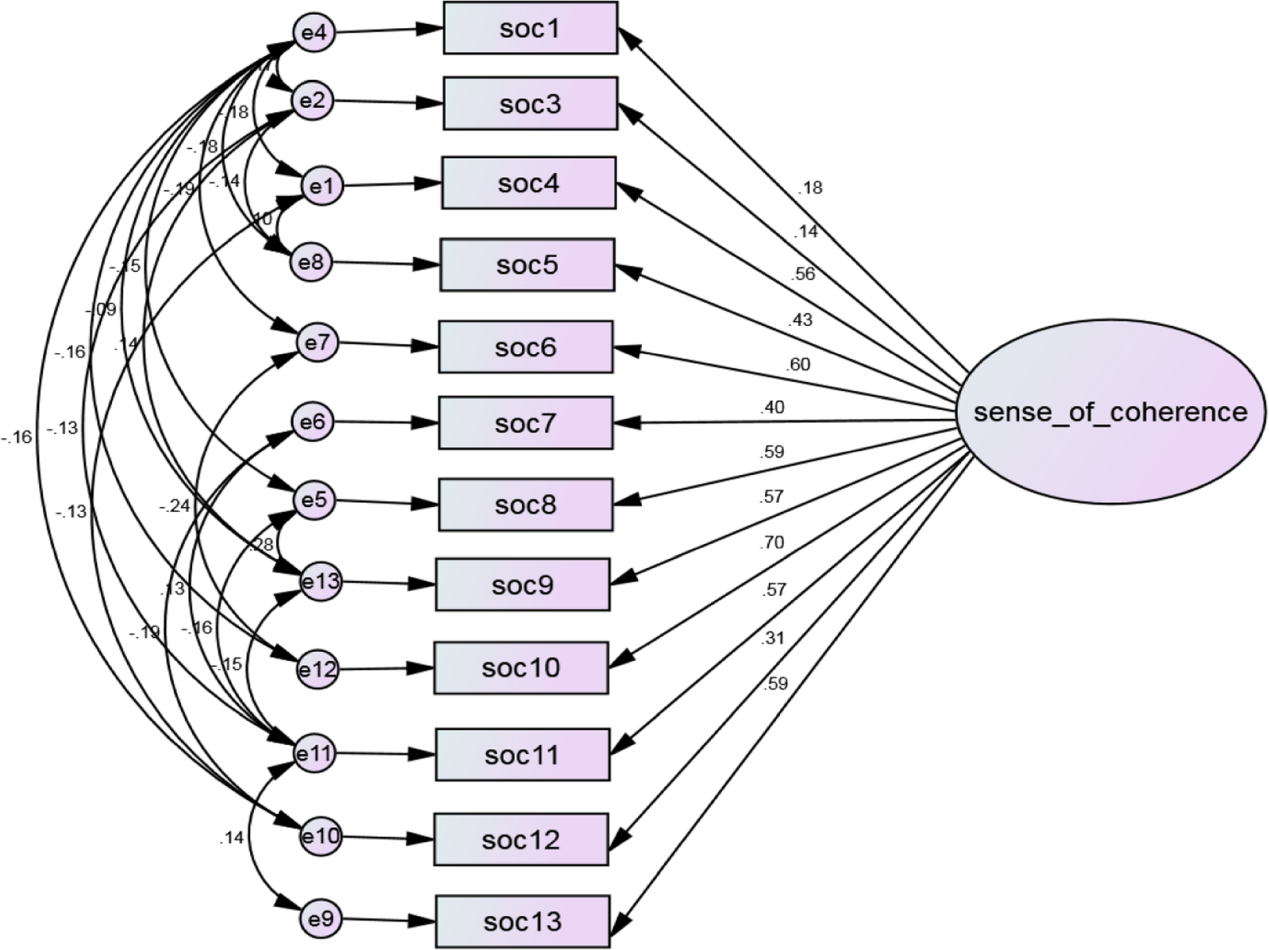
One factor model of the Tigrigna version of SoC-12, with correlated error terms. Rectangles represent indicator items; ovals represent latent factors; single headed arrows along with standardized weights represent factor loadings; circles represent error terms for each item (e), and disturbance terms of each latent factor (d); SoC=Sense of Coherence. Model fit: χ^2^ = 57.54; χ^2^/df = 1.692; CFI = 0.982; TLI = 0.964; SRMR = 0.0280; RMSEA = 0.035

Single factor structure of SoC-13, uncorrelated error terms, poorly fitted the data (CFI < 0.95; RMSEA> 0.05) (see Fig. 2). However, after re-specification of the model following modification index (MI), allowing error terms to correlate and trimming item-2 with insignificant loading  β= 0.062, *p* > 0.05), the one factor structure of SoC with twelve items best fitted the present data compared to other plausible models tested (see Fig. 3).

Of all models tested (Table 2), the one latent factor structure of So-C with 12-items best fit the present data after allowing error terms to correlate (CFI = 0.982; RMSEA = 0.035).

**Table 2:** Comparison of fit indices for computing factor structures of SoC-13 and their modifications in Eritrean refugees living in Ethiopia

Proposed Models for SoC-13	χ^2^	χ^2^/df	CFI	GFI	TLI	SRMR	RMSEA	*P*-value
1. Single factor SoC-13 model, uncorrelated error terms	412.363	6.34	0.752	0.889	0.703	0.079	0.107	*p* < 0.001
2. Single factor SoC-13 model with correlated error terms (SoC-13)	110.31	2.40	0.954	0.971	0.922	0.043	0.050	*p* < 0 .001
3. Single factor model with correlated error terms, modified (SoC-12)	57.54	1.692	0.982	0.983	0.964	0.028	0.035	*P* < 0.01
4. Three correlated factors (SoC-13), correlated error terms	356.54	6.368	0.786	0.912	0.702	0.093	0.098	*p* < 0.001

Legend: χ^2^ = Chi-square; χ^2^/df = Chi-square to degree of freedom; *CFI* Comparative Fit index; GFI = Goodness of Fit

Index; *TLI* Tucker Lewis Index; *SRMR* Standardized Root Mean Residual; *RMSEA* Root Mean Square

Error of Approximations

### Convergent validity

**Fig. 4:**
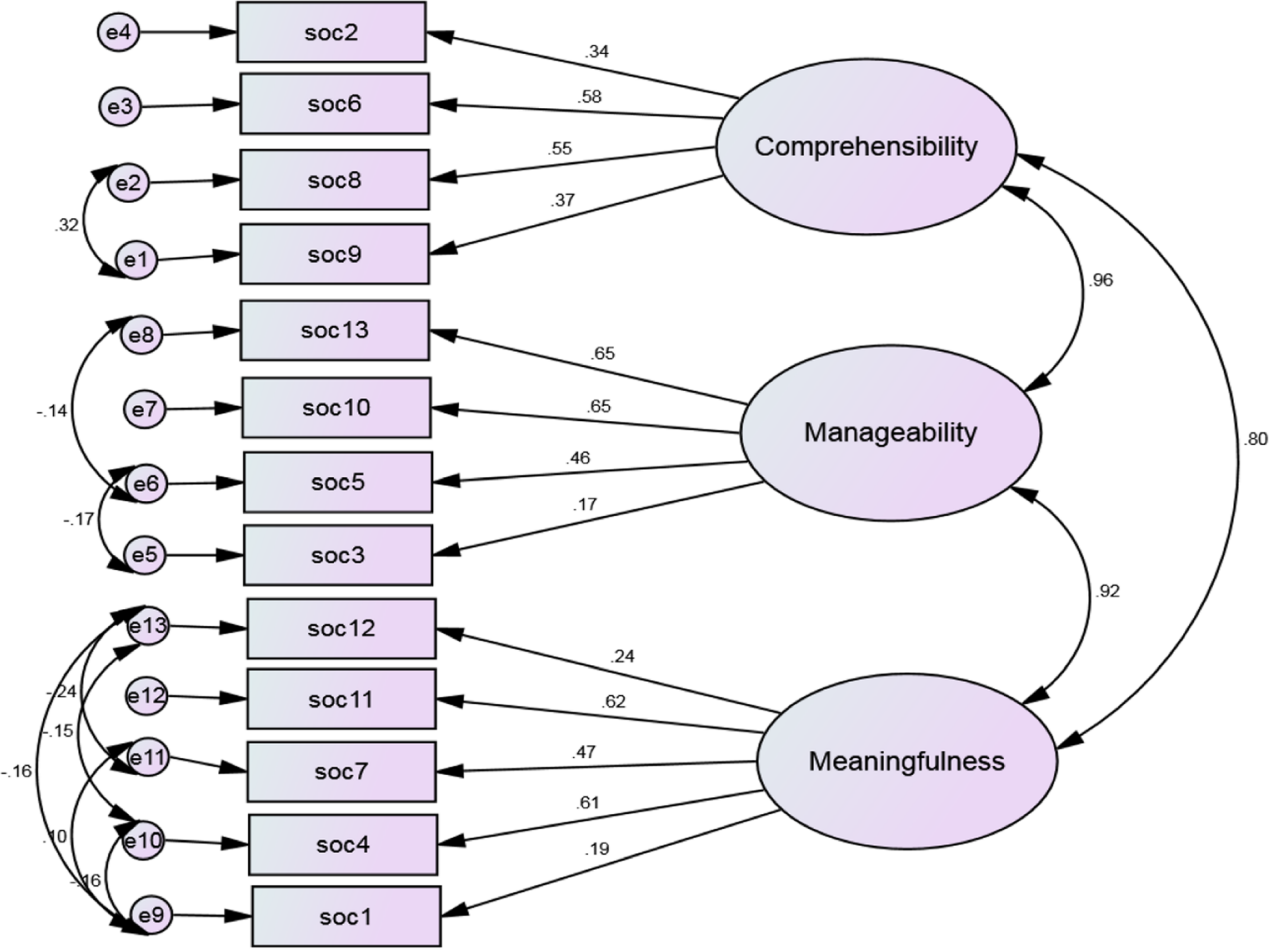
Three correlated factors of the Tigrigna version of SoC-13, with correlated error terms. Rectangles represent indicator items; ovals represent latent factors; single headed arrows along with standardized weights represent factor loadings; double headed arrows (right) represent co-variances between latent factors; double headed arrows (left) represent co-variances between factors; circles represent error(disturbance) terms for each item; SoC=Sense of Coherence. Model fit*:* χ^2^ = 356.54; χ^2^/df = 6.368; CFI = 0.786; TLI = 0.702; SRMR = 0.0928; RMSEA = 0.098

As indicated in Table 3, twelve items have significantly loaded onto a single latent factor, with standardized path coefficients ranging from 0.14 (item-3) to 0.70 (item-10) in a single factor model of soC-13. When evaluated for the strength and adequacy of item loadings, Item-1, item-2, item-3 and item-12 are weak items, because they demonstrated poor convergent validity (β < 0.40) to the expected latent factor. A comparison of item loadings regarding the thirteen items of SoC-13 of the Eritrean refugee sample in the current study with findings of other previous studies using CFA analysis in different cultural settings was done, and the findings  are summarized in Table 3. In the three factors model of SoC-13 (Fig. 4), however, all the thirteen items significantly loaded onto their respective factors although the model demonstrated poor fit. In addition, there is a significant positive correlation of SoC-13 to related measures; specifically, SoC-13 is positively and significantly correlated with Oslo Social Support Scale (OSS-3) r = 0.363 *p* < .001. SoC-13 has also demonstrated weak, but positive association with task-oriented coping (r = 0.089, *p* < 0.001).

**Table 3:** Comparison of item loadings of each item of SoC-13 in Eritrean sample with previous evidence

Author	SoC-13 Items		1	2	3	4	5	6	7	8	9	10	11	12	13	Best fitting structure of SoC-13
Present study	Eritrean refugees (n = 562)	1 factor	0.18	n.s.	0.14	0.56	0.43	0.60	0.40	0.59	0.57	0.70	0.57	0.31	0.59	One factor SoC-12, correlated error terms (CFI = 0.98; RMSEA = 0.035)
		3 factors	0.19	0.34	0.17	0.61	0.46	0.58	0.47	0.55	0.37	0.65	0.62	0.24	0.65	
Bonacchi, etal.(2012)	*Italian sample(*n* = 372)	1 factor	0.30	0.28	0.37	0.48	0.41	0.60	0.43	0.67	0.50	0.55	0.30	0.62	0.66	One factor SoC-13, correlated error terms (CFI = 0.93; RMSEA = 0.05)
LuyckxK, et al.(2012)	*Dutch speaking Belgians (*n* = 2781)	Three factors	0.38	n.s.	n.s.	0.31	0.28	0.43	0.52	0.76	0.76	0.79	0.60	0.32	0.69	First–order three correlated factors model & second-order model of SoC-11 (CFI = 0.93; RMSEA = 0.06)
Saravia, et al.(2014)	*Peruvian Sample (*n* = 448)	1 factor	0.35	0.27	0.37	0.44	0.36	0.47	0.40	0.68	0.70	0.61	0.37	0.66	0.61	Three correlated model of soc-13 (CFI = 0.92; RMSEA = 0.06)
		3 factors	0.38	0.29	0.41	0.50	0.38	0.48	0.50	0.69	0.72	0.63	0.36	0.77	0.64	

n.s. = not significant (items are discarded if not significant)

*Saravia JC, Iberico C, Yearwood K: Validation of Sense of Coherence (soc) 13-item scale in a Peruvian Sample. *Journal of Behavior, Health & Social Issues* 2014; 6(2): 35–44

*Bonacchi, A., Miccinesi, G., Galli, S., Chiesi, F., Martire, M., Guazzini, M., et.al. The dimensionality of the Sense of Coherence Scales*,* an investigation on Italian sample. *TPM 2012; 19(2):* 115–134

*Luyckx K, Goossens E, Apers S, et al.: The 13-Item Sense of Coherence scale in Dutch-speaking Adolescents and young adults: Structural validity, age trends, and chronic disease. *Psychologica Belgica* 2012; 52(4): 351–368

### Divergent validity

The bi-variate analysis Pearson’s correlation (r) between SoC-13, and different constructs measuring adverse conditions, demonstrated an association to the expected direction. Thus, the association between SoC-13 and measures for adversities is inverse and significant. Specifically, SoC-13 is inversely and significantly related to PC-PTSD (r = − 0. 433, *p* < 0.001), CES-D (r = − 0.597, p < 0.001), pre-migration living difficulties (r = − 0.265, *p* < 0.001), and post-migration living difficulties(r = − 0.249, *p* < .001). It also demonstrated significant but weak negative association with FAST (r = − 0.105, *p* < 0.001).

### Discriminant validity

Examination of the co-variances between the three latent factors of SoC-13 indicate that the standardized coefficients for the three latent factors were found greater than or equal to 0.80 (above the maximum cut-off point for correlation coefficient to discriminate between factors) (Fig. 4).

## Discussion

Unmodified measure of sense of coherence with thirteen items (SoC-13) has demonstrated acceptable convergent as well as divergent validity. However, when evaluated the overall fit of the unmodified model (SoC-13), with uncorrelated error terms, it demonstrated poor fit to the present data (CFI < 0.95; TLI < 0.95 and RMSEA > 0.08) compared to the fit indices of the same scale with specified model in samples from South Africa [54].

After permission of error terms to correlate, the present data fit best with single latent factor model of Sense of Coherence scale with twelve items (SoC-12) significantly loaded onto a single latent factor (χ^2^ = 57.54, df = 24; χ^2^/df = 1.69; CFI = 0.98; RMSEA = .035) compared to other computing models of SoC-13 tested based on relevant literature. Hence our data supported the single factor structure of the short form of SoC proposed by the original scale developer [34]. However, when the thirteen items were evaluated for the strength of their loadings, item-1, item-2, item-3 and item-12 demonstrated poor loadings (β < 0.40), and item-2 demonstrated non-significant loading (β = 0.06, p>0.05).

Hence, the internal consistency as measured by Cronbach’s alpha remain stable, even after deleting item-2, and changed from 0.736 for SoC-13 to a slight improvement of 0.748 for SoC-12. Therefore, item- 2 which reads: “*Has it happened in the past that you were surprised by the behavior of people whom you thought you knew well*?” (see Additional file 1: Table S3) is not valid to Eritrean culture in its current form of presentation, because it has demonstrated very low item loading that doesn’t reach significance level. Similarly, this item was also found to have relatively lower item loadings (β<0.40) as well as considered as problematic items in previous studies done in different cultural contexts and populations [6, 16, 20]. For example, item-2 demonstrated insignificant loadings to a three latent factor model of SoC-11, which fit data for a Dutch speaking Beligian sample [6]. For this particular item, the intact rich social connectedness among Eritreans, being from a collectivist society, may explain why respondents may not have understood the item as part of their concern.

The current findings with respect to lower item loadings of item-1 can be justified by evidence from previous qualitative investigation of how displaced Eritreans reacted to each items of sense of coherence [2]. Their findings indicate that Eritrean participants reacted to item-1: “*do you have feeling that you don’t really care about what goes on around you?*” by responding to the question itself with feelings of surprise saying: “*how can you ask such a question? I wouldn’t be human if I didn’t care about what goes on around me…, this is not in our culture … of course, I care and try to do something about it, if I can*.”(Alemdom,2007; p.97) [2]. It means Eritreans value a strong social life such that life for them is entirely interdependent in contrast to people from western cultural background, who may have grown up in a culture where indivudualistic culture is so deeply rooted. For item-3, which reads: “*Has it happened that people whom you counted on disappointed you?”* the relatively lower item loading onto a single latent factor has a similar justification as in the case for item-1. Hence the deep rooted trust in others, as a matter of a shared cultural norm for Eritreans, may best justify the lower convergent validity of this item too. However, the weaker item loadings of the first three items seen in Eritrean refugee sample in the present study are not unique to the type of population as well as study context. Cross comparison of item loadings across studies for the observed weaker loadings of item-1, item-2 and item-3 indicated that these items also demonstrated relatively weaker convergent validity (weaker loadings of β<0.40) in previous studies conducted in western settings, such as CFA studies conducted in Italy [16], Belgium [6] as well as a non-western setting, such as Peru [20].

Attaining a better fit for a model through model modification and omission of an item or a few items is also a practice of amending measures to be valid in studies conducted in different cultural settings [6, 13, 19, 53, 54]. For example, only 11 items from the Sense of Coherence scale were suggested as justifiable measures of capturing what SoC-13 can perform in Dutch speaking Belgians, after disregarding the two items (i.e. item-2 and item-3) having insignificant loadings [6] (see Table 3). Similar to the Tigrigna version of SoC-12 model with correlated error terms in the present study, error terms were also allowed to correlate in a one factor model of SoC-13 suggested as best fit to data of an Italian sample [16].

However, when comparison of the current data with previous studies done across cultures was made, differences were observed on the strength of loadings for item-12, which asks: “*How often do you have the feeling that there’s little meaning in the things you do in your daily life*?”. The item loading for this item onto both the one factor as well as the three factors models of SoC-13 is poor (β < 0.40) in Eritrean sample in the current study, while this item sufficiently loaded onto one factor as well as three factors in the Peruvian sample [20] (see Table 3).

With respect to factor structure, the present CFA finding is not in line with previous findings, which supported a three factor model of SoC-13 in old samples of Netherlands [18], patients with morbid obesity [55], and Dutch speaking Belgians [6]. The present study supported a previous study in an Italian sample, whose data best fit a one factor model of SoC-13 [16]. Despite the conflicting findings regarding the dimensionality of SoC-13, the data in the present study for Eritrean refugee sample best fit with one factor structure of sense of coherence scale with 12 items (CFI =0 .982, RMSEA = 0.035 [90%CI = 0.018,0.050], which supported the factor structure proposed by the original scale developer, Antonovsky [34].

The fact that the present study demonstrated very high co-variances among the three correlated latent factors (r ≥ 0.80, *p* < 0.001) (Fig. 4) may serve as additional evidence to propose that the factors seem to measure similar or same construct in the context of Eritrean refugees living in Ethiopia.

The mean value in the current study suggested that individuals’ sense of coherence is compromised by being a refugee (mean = 39.91) compared to those internal displaced persons as well as non-displaced people whose mean were reported to be 48.94 and 54.84 respectively in the previous study [2] (see Additional file 4: Table S4).

The internal consistency for SoC-13 (Cronbach’s alpha = 0.74) as well as the internal consistency for SoC-12 (Cronbach’s alpha = 0.75) is high, and these are within the range of alpha values reported (i.e. 0.74 to 0.91) [1]. The current alpha coefficient is also in line with a study involving systematic review of 127 studies, which reported the reliability range from 0.70 to 0.92 [17]. Reliability coefficients which range from 0.70 to 0.90 are demonstrating high reliability [56].

## Strengths and limitations of the study

Given that there is a paucity of locally adapted measures to measure resilience factors for Eritreans in humanitarian settings, the present measurement study, following rigorous procedures of adaptation filled gaps noted in the previous studies. Making use of a comparative analysis with other previous CFA studies done across cultures with findings of the present study can also be taken as the strength of the study. A comparison of data across studies gives the reader a clearer picture of contrast to the relative relevance of items with weaker loadings across cultures and type of population. However, caution should be taken while making a comparison of findings obtained from the 5-point response format of the Tigrigna version of SoC-13 in the present study with other findings which used a 7- point response format of the same scale in other cultural contexts. Another limitation of the present study is that we did not conduct a sub-sample analysis which would have permitted us to see if the factor structure is confounded by some basic demographic variables, such as age and gender. This study would also have profited if data from comparable groups of sample had been collected, because it would have strengthened the external validity of the findings.

## Conclusions, implications for clinical practice and future direction

The short form of SoC with twelve items seems an appropriate measure of sense of coherence for Eritrean refugees living in Ethiopia, which should be understood as a uni-dimensional construct. Therefore, the Tigrigna version of sense of coherence, with 12-items is a valid measure with its acceptable internal consistency. Additional inference derived from covariance of the three theoretical latent factor structures (r ≥ 0.80), which demonstrated above the maximum cut-off point for discriminant validity, implies the likelihood of the present data to support a single factor structure of the Tigrigna version of sense of coherence as a more reasonable factor structure compared to a three factor structure to the Eritrean cultural context. Omission of item-2 substantially improved fit indices as well as item loadings for other items. Hence the reduced Tigrigna version SoC-12 is a good measure for assessing resilience and can be taken as a proxy measure of mental wellbeing for Eritreans living in Ethiopian emergency settings. It can be employed by psychiatrists, counselors, social workers, and researchers in clinical as well as non-clinical settings for assessing resilience for the ultimate purpose of generating data helpful to make an informed decision in primary mental health care for the community-based psychosocial intervention as well as counseling. It may also provide a supplementary source of information for clinical decision making.

Future studies should undertake a qualitative study on the phenomenology of sense of coherence in Eritrean communities so that Antonovsk’s problematic items in the Tigrigna version will be further improved and adapted using valid concepts from Eritrean culture. Hence adaptation should consider their frame of understanding to the holistic and overall Eritrean way of life style, including their inner psyche, spiritual, social, familial and community life, their collective culture, trust, hope, tradition and their belief in rituals, etc. In addition, future clinical practice and interventions regarding refugee mental health in humanitarian settings of Africa, like in refugee camps of Ethiopia for Eritreans, should be geared towards alternative use of this measure to assess mental wellbeing rather than being confined solely on the assessment and diagnosis of pathology. Unfortunately this trend of measuring pathology only is primarily practiced as the predominant means of assessment in mental healthcare practices by humanitarian institutions and practitioners working to assist the mental health of refugees or displaced people in such settings. In order for the current findings to be replicable, future research should be carried out using longitudinal study designs. Furthermore CFA study on the validity of sense of coherence based on data from multiple samples of Eritreans is needed in the future to fairly generalize the factorial structure and construct validity of this tool in Eritrean culture.

## Additional files

Additional file 1: **Table S3.** The 13-items of Sense of Coherence (SoC-13) scale with five point response format, adapted for Eritrean refugees (DOCX 14 kb)

Additional file 2: **Table S1.** Internal consistency of the three sub-scales of SoC-13 in Eritrean refugees living in Ethiopia. (DOCX 17 kb)

Additional file 3: **Table S2.** Item- total correlation and Item level Content Validity Index(I-CVI) for each items of SoC-13 scale (DOCX 14 kb)

Additional file 4: **Table S4.** Mean score comparison of SoC-13 between two studies from Eritrean sample. (DOCX 17 kb)

## Abbreviations

AAU: Addis Ababa University
ARRA: Administration of Refugee and Returnees Affairs
CES-D: Center for Epidemiologic Studies Depression Scale
CFI: Comparative Fit Index
CVT: Center for Victims of Trauma
I-CVI: Item-Level Content Validity Index
IRC: International Rescue Committee
JRS: Jesuit Refugee Service
NRC: Norwegian Refugee Council
OSS-3: Oslo Social Support Scale,3- Items
PC-PTSD: Primary Care PTSD Screener
RMSEA: Root Mean Square Error of Approximation
S-CVI/Ave: Scale-Level Content Validity Index, Average method
SoC-13: Sense of Coherence Scale,13-Items
SRMR: Standardized Root Mean Square Residual
TLI: Tuker Lewis Index
UNHCR: United Nations Higher Commissioner for Refugees
