# Supplementary material for: Construct validity and factor structure of sense of coherence (SoC-13) scale as a measure of resilience in Eritrean refugees living in Ethiopia
Source: Confl Health. 2019 Feb 6;13:3. doi: 10.1186/s13031-019-0185-1 (PMC6366046; doi:10.1186/s13031-019-0185-1)
Supplement: Supplementary file 1 — Table S3. The 13-items of Sense of Coherence (SoC-13) scale with five point response format, adapted for Eritrean refugees (DOCX 14 kb) [file 13031_2019_185_MOESM1_ESM.docx]

Supplement Table-3: The 13-items of Sense of Coherence (SoC-13) scale with five point response format, adapted for Eritrean refugees

| \| 1. *Do you have feeling that you don’t really care about what goes on around you?   \| 1 \| 2 \| 3 \| 4 \| 5 \|  \|  \| \| --- \| --- \| --- \| --- \| --- \| --- \| --- \|   (very seldom or never) (very often) \| \| --- \| --- \| --- \| --- \| --- \| --- \| --- \| --- \| \| 2. *Has it happened in the past that you were surprised by the behavior of people whom you thought you knew well?   \| 1 \| 2 \| 3 \| 4 \| 5 \|  \|  \| \| --- \| --- \| --- \| --- \| --- \| --- \| --- \|   (never happened) (always happened) \| \| 3.* Has it happened that people whom you counted on disappointed you?   \| 1 \| 2 \| 3 \| 4 \| 5 \|  \|  \| \| --- \| --- \| --- \| --- \| --- \| --- \| --- \|   (never happened) (Always happened) \| \| 4. Until now your life has had:   \| 1 \| 2 \| 3 \| 4 \| 5 \|  \|  \| \| --- \| --- \| --- \| --- \| --- \| --- \| --- \|   (No clear goals  or purpose at all) (very clear goals and purpose) \| \| 5. Do you have the feeling that you’re being treated unfairly?   \| 1 \| 2 \| 3 \| 4 \| 5 \|  \|  \| \| --- \| --- \| --- \| --- \| --- \| --- \| --- \|   (very often) (very seldom or never) \| \| 6. Do you have the feeling that you are in an unfamiliar situation and don’t know what to do?   \| 1 \| 2 \| 3 \| 4 \| 5 \|  \|  \| \| --- \| --- \| --- \| --- \| --- \| --- \| --- \|   (very often) (very seldom or never) \| \| 7.* Doing the thing you do every day is:   \| 1 \| 2 \| 3 \| 4 \| 5 \|  \|  \| \| --- \| --- \| --- \| --- \| --- \| --- \| --- \|   (A source of deep (a source of pain and boredom)  pleasure and  satisfaction) \| \| 8. Do you have very mixed-up feelings and ideas?   \| 1 \| 2 \| 3 \| 4 \| 5 \|  \|  \| \| --- \| --- \| --- \| --- \| --- \| --- \| --- \|   (very often) (very seldom or never) \| \| 9. Does it happen that you have feelings inside you would rather not feel?   \| 1 \| 2 \| 3 \| 4 \| 5 \|  \|  \| \| --- \| --- \| --- \| --- \| --- \| --- \| --- \|   (very often) (very seldom or never) \| \| 10.* Many people – even those with a strong character – sometimes feel like sad sacks (losers) in certain situations. How often have you felt this way in the past?   \| 1 \| 2 \| 3 \| 4 \| 5 \|  \|  \| \| --- \| --- \| --- \| --- \| --- \| --- \| --- \|   (Never) (very often) \| \| 11. When something happened, have you generally found that:   \| 1 \| 2 \| 3 \| 4 \| 5 \|  \|  \| \| --- \| --- \| --- \| --- \| --- \| --- \| --- \|   (you overestimated (you saw in the right proportion)  or understand  its importance) \| \| 12. How often do you have the feeling that there’s little meaning in the things you do in your daily life?   \| 1 \| 2 \| 3 \| 4 \| 5 \|  \|  \| \| --- \| --- \| --- \| --- \| --- \| --- \| --- \|   (very often) (very seldom or never) \| \| 13. How often do you have feelings that you’re not sure you can keep under control?   \| 1 \| 2 \| 3 \| 4 \| 5 \|  \|  \| \| --- \| --- \| --- \| --- \| --- \| --- \| --- \|   (very often) (very seldom ) \| |
| --- | --- | --- | --- | --- | --- | --- | --- | --- | --- | --- | --- | --- | --- | --- | --- | --- | --- | --- | --- | --- | --- | --- | --- | --- | --- | --- | --- | --- | --- | --- | --- | --- | --- | --- | --- | --- | --- | --- | --- | --- | --- | --- | --- | --- | --- | --- | --- | --- | --- | --- | --- | --- | --- | --- | --- | --- | --- | --- | --- | --- | --- | --- | --- | --- | --- | --- | --- | --- | --- | --- | --- | --- | --- | --- | --- | --- | --- | --- | --- | --- | --- | --- | --- | --- | --- | --- | --- | --- | --- | --- | --- | --- | --- | --- | --- | --- | --- | --- | --- | --- | --- | --- | --- | --- |

* = reverse score
