# Supplementary material for: Construct validity and factor structure of sense of coherence (SoC-13) scale as a measure of resilience in Eritrean refugees living in Ethiopia
Source: Confl Health. 2019 Feb 6;13:3. doi: 10.1186/s13031-019-0185-1 (PMC6366046; doi:10.1186/s13031-019-0185-1)
Supplement: Supplementary file 2 — Table S1. Internal consistency of the three sub-scales of SoC-13 in Eritrean refugees living in Ethiopia. (DOCX 17 kb) [file 13031_2019_185_MOESM2_ESM.docx]

Suppliment Table-1: Internal consistency of the three sub-scales of SoC-13 in Eritrean refugees living in ethiopia

| \| Sub-scales of SoC-13 \| Cronbach's Alpha \| \| Items \| No of items \| \| --- \| --- \| --- \| --- \| --- \| \|  \| Pilot test(n=52) \| Main study(n=562) \|  \|  \| \| Comprehensibility \| 0.605 \| 0.563 \| 2,6,8,9 \| 4 \| \| Manageability \| 0.480 \| 0.459 \| 3,5,10,13 \| 4 \| \| Meaningfulness \| 0.160 \| 0.397 \| 1,4,7,11,12 \| 5 \| \| Total scale \| 0.668 \| 0.736 \| 1-13 \|  \| |
| --- | --- | --- | --- | --- | --- | --- | --- | --- | --- | --- | --- | --- | --- | --- | --- | --- | --- | --- | --- | --- | --- | --- | --- | --- | --- | --- | --- | --- | --- | --- |
