# Supplementary material for: Construct validity and factor structure of sense of coherence (SoC-13) scale as a measure of resilience in Eritrean refugees living in Ethiopia
Source: Confl Health. 2019 Feb 6;13:3. doi: 10.1186/s13031-019-0185-1 (PMC6366046; doi:10.1186/s13031-019-0185-1)
Supplement: Supplementary file 3 — Table S2. Item- total correlation and Item level Content Validity Index(I-CVI) for each items of SoC-13 scale (DOCX 14 kb) [file 13031_2019_185_MOESM3_ESM.docx]

Supplement Table-2: item- total correlation and Item level Content Validity Index( I-CVI) for each items of SoC-13 scale

| \| \| Items \| \| \| 1 \| 2 \| 3 \| 4 \| 5 \| 6 \| 7 \| 8 \| 9 \| 10 \| 11 \| 12 \| 13 \| \| --- \| --- \| --- \| --- \| --- \| --- \| --- \| --- \| --- \| --- \| --- \| --- \| --- \| --- \| --- \| --- \| \| Item- total  Correlation \| Pilot study (n=52) \| \| 0.27 \| 0.32 \| 0.37 \| 0.53 \| 0.48 \| 0.65 \| 0.41 \| 0.50 \| 0.58 \| 0.63 \| 0.45 \| 0.10 \| 0.52 \| \| Main study(n=562) \| \| 0.24 \| 0.26 \| 0.36 \| 0.57 \| 0.45 \| 0.58 \| 0.47 \| 0.64 \| 0.65 \| 0.66 \| 0.56 \| 0.33 \| 0.63 \| \| Alpha if item deleted \| Main study(n=562) \| \| 0.76 \| 0.75 \| 0.73 \| 0.71 \| 0.72 \| 0.71 \| 0.72 \| 0.70 \| 0.70 \| 0.70 \| 0.71 \| 0.74 \| 0.70 \| \| I-CVI \| \| Main study (n=562) \| 1 \| 1 \| 1 \| 1 \| 0.86 \| 1 \| 1 \| 1 \| 1 \| 1 \| 1 \| 1 \| 1 \| \| \| --- \| --- \| --- \| --- \| --- \| --- \| --- \| --- \| --- \| --- \| --- \| --- \| --- \| --- \| --- \| --- \| --- \| --- \| --- \| --- \| --- \| --- \| --- \| --- \| --- \| --- \| --- \| --- \| --- \| --- \| --- \| --- \| --- \| --- \| --- \| --- \| --- \| --- \| --- \| --- \| --- \| --- \| --- \| --- \| --- \| --- \| --- \| --- \| --- \| --- \| --- \| --- \| --- \| --- \| --- \| --- \| --- \| --- \| --- \| --- \| --- \| --- \| --- \| --- \| --- \| --- \| --- \| --- \| --- \| --- \| --- \| --- \| --- \| --- \| --- \| --- \| --- \| --- \| --- \| --- \| |
| --- | --- | --- | --- | --- | --- | --- | --- | --- | --- | --- | --- | --- | --- | --- | --- | --- | --- | --- | --- | --- | --- | --- | --- | --- | --- | --- | --- | --- | --- | --- | --- | --- | --- | --- | --- | --- | --- | --- | --- | --- | --- | --- | --- | --- | --- | --- | --- | --- | --- | --- | --- | --- | --- | --- | --- | --- | --- | --- | --- | --- | --- | --- | --- | --- | --- | --- | --- | --- | --- | --- | --- | --- | --- | --- | --- | --- | --- | --- | --- | --- |

Abbreviations: Item level Content Validity index(I-CVI)=.86-1 ;CVI-S-Scale level Content Validity Index (S-CVI)= 0.92,

Average Scale- Level Content Vaidity Index (S-CVI/Ave) = 0.989
