# Supplementary material for: Construct validity and factor structure of sense of coherence (SoC-13) scale as a measure of resilience in Eritrean refugees living in Ethiopia
Source: Confl Health. 2019 Feb 6;13:3. doi: 10.1186/s13031-019-0185-1 (PMC6366046; doi:10.1186/s13031-019-0185-1)
Supplement: Supplementary file 4 — Table S4. Mean score comparison of SoC-13 between two studies from Eritrean sample. (DOCX 17 kb) [file 13031_2019_185_MOESM4_ESM.docx]

Table-5: Mean score comparison of SoC-13 between two studies from Eritrean sample

|  |  |  |
| --- | --- | --- |
| \| Study \| Participants \| Sample size \| Mean(SD) \| Median \| Range \| Reliability  Alpha \| Split half reliability \| \| --- \| --- \| --- \| --- \| --- \| --- \| --- \| --- \| \| Present  Study \| Eritrean  Refugees in Ethiopia \| Total sample  (n=562) \| 38.91(8.66) \| 38 \| 15- 63 \| 0.74 \| 0.69 \| \| Male(n=258) \| 39.89(8.73) \| 40 \| 18-60 \| 0.72 \| 0.71 \| \| Female(n=304) \| 38.08(8.53) \| 37.5 \| 15-63 \| 0.75 \| 0.66 \| \| Alemdom, et al.,2007* \| Eritrean  IDP \| Total sample  (n=72) \| 48.94 \|  \|  \|  \|  \| \| Male(n=40) \| 52.5 \|  \|  \|  \|  \| \| Female(n=32) \| 41.18 \|  \|  \|  \|  \| \| Eritrean  Non-displaced \| Total sample  (N=193) \| 54.84 \|  \|  \|  \|  \| \| Male(n=63) \| 53.61 \|  \|  \|  \|  \| \| Female(n=130) \| 54.66 \|  \|  \|  \|  \| \|  \|  \|  \|  \|  \|  \|   Note: IDP (Internally Displaced Persons); shaded= not reported  *Almedom A, Tesfamichael B, Saeed Z, et al: use of ‘sense of coherence (SoC)’ scale to measure resilience in Eritrea: Interrogating both the data and the scale. J.biosoc.Sci, 2007; 39, 91–107 |  |  |
